# Everything You Always Wanted to Say about Science (But Were Afraid to Publish)

**DOI:** 10.1523/ENEURO.0115-22.2022

**Published:** 2022-03-28

**Authors:** Christophe Bernard

As you know, the raison d’être of *eNeuro* is to serve the neuroscience community. The main mission of the journal is to publish excellent and reliable results, while making sure that authors, reviewers, and reviewing editors have a great experience. But *eNeuro* offers other services to the community, as well: it is a forum open to all if the message you want to convey can help us neuroscientists, teach us something, question us, or make us reflect on important matters.

The present state of neuroscience is not only the result of the knowledge that is being accumulated over time but also strongly influenced by multiple factors. These factors include how the scientific method is taught in courses and passed over to the young generations in laboratories, prevailing dogmas that can prevent the emergence of alternative hypotheses and new ideas, and the pressure to publish “high” at all costs to obtain a position or a grant.

*eNeuro* includes two article types, commentaries and opinions, which are there for you to express yourself. We have a very successful series of these papers called “Experimental Bias” (https://www.eneuro.org/collection/experimental-bias), which alerts us to the problems of interpretation of several experimental paradigms or to what a given technique can really tell. Another recent commentary discussed how a common fallacy may have led the field of Alzheimer’s disease astray (Herrup, 2022).

A common reaction to such pieces is “this has been discussed a century ago,” “this is already known,” etc. But the mere fact that some ideas need to be repeated only means that they are not understood or integrated by individuals, and more importantly by the upcoming generation. Fallacies were identified and listed by Aristotle >2300 years ago. No matter how known the phenomenon is, since we are still falling in the fallacy trap, the message will need to be repeated over and over.

Do not censor yourself. If you want to alert us, tell us something that educates us, show us where we may be mistaken, and in which direction we should go, *eNeuro* is here for you. It does not matter if the topic has been covered already. What is important is to propose solutions, to help us make better science.

This is for the “science” side of it. Science does not exist for and by itself. It is part of the society. Thus, societal issues are as important to discuss. To name a few: parity (in the most general sense), social media, funding agencies, or contribution to global warming when doing science.

The format is flexible. It can be a standalone piece, a dialogue, such as the very successful dialogue between Buzsáki (2020) and David Poeppel (Poeppel and Adolfi, 2020), or a collection of pieces, like *eNeuro*’s collection on the Blue Brain Project (https://www.eneuro.org/collection/epistemological-lessons).

If you want your message to be heard, *eNeuro* will be your vehicle, the way you want it to be.
